# Effect of standardized vs. local preoperative enteral feeding practice on the incidence of NEC in infants with duct dependent lesions: Protocol for a randomized control trial

**DOI:** 10.3389/fcvm.2022.893764

**Published:** 2022-09-08

**Authors:** Joanna Seliga-Siwecka, Ariel Płotko, Agata Wójcik-Sep, Renata Bokiniec, Julita Latka-Grot, Małgorzata Żuk, Konrad Furmańczyk, Wojciech Zieliński, Mariola Chrzanowska

**Affiliations:** ^1^Department of Neonatology and Neonatal Intensive Care, Medical University of Warsaw, Warsaw, Poland; ^2^Department of Neonatology, Children's Health Memorial Institute, Warsaw, Poland; ^3^Cardiology Clinic, Children's Health Memorial Institute, Warsaw, Poland; ^4^Department of Applied Mathematics, Institute of Information Technology, Warsaw University of Life Sciences, Warsaw, Poland; ^5^Department of Prevention of Environmental Hazards, Allergology and Immunology, Medical University of Warsaw, Warsaw, Poland; ^6^Department of Statistics and Econometrics, Institute of Economics and Finance, Warsaw University of Life Sciences, Warsaw, Poland

**Keywords:** congenital heart defect, enteral feeding algorithm, intensive care unit, neonate, duct dependant

## Abstract

**Background:**

Infants with duct dependent heart lesions often require invasive procedures during the neonatal or early infancy period. These patients remain a challenge for pediatric cardiologists, neonatologists, and intensive care unit personnel. A relevant portion of these infant suffer from respiratory, cardiac failure and may develop NEC, which leads to inadequate growth and nutrition, causing delayed or complicated cardiac surgery.

**Methods:**

This randomized control trial will recruit term infants diagnosed with a duct dependant lesion within the first 72 h of life. After obtaining written parental consent patients will be randomized to either the physician led enteral feeding or protocol-based feeding group. The intervention will continue up to 28 days of life or day of cardiosurgical treatment, whichever comes first. The primary outcomes include NEC and death related to NEC. Secondary outcomes include among others, number of interrupted feedings, growth velocity, daily protein and caloric intake, days to reach full enteral feeding and on mechanical ventilation.

**Discussion:**

Our study will be the first randomized control trial to evaluate if standard (as in healthy newborns) initiation and advancement of enteral feeding is safe, improves short term outcomes and does not increase the risk of NEC. If the studied feeding regime proves to be intact, swift implementation and advancement of enteral nutrition may become a recommendation.

**Trial registration:**

The study protocol has been approved by the local ethical board. It is registered at ClinicalTrials.gov NCT05117164.

## Introduction

### Background and rationale

Congenital heart diseases (CHD) occur with an incidence of six to nine cases per 1,000 births (1). Within this group, infants with duct dependent heart lesions often require invasive procedures during the neonatal or early infancy period. Furthermore, a relevant portion of these infants suffer from respiratory and cardiac failure as well as necrotizing enterocolitis (NEC) which leads to malnutrition and failure to thrive within the first months of life (2, 3). Inadequate growth and nutrition often lead to delayed or complicated cardiac surgery (4). NEC is a very rare condition in full-term neonates, however the risk increases by 10–100 times in patients with CHD (2). The estimated rate of NEC in infants with CHD ranges from 1.6 to 9% and mortality rates go up to 24% (5). The exact pathophysiology of NEC in babies w CHD is unknown but is likely multifactorial. Impaired mesenteric blood flow seen in infants with CHD is caused by the disruption of systemic perfusion during diastole and predisposes the infant to NEC (6, 7). McElhinney et al. have shown that episodes of low cardiac output and shock were significantly associated with NEC with an odd ratio of 6.5 (95% CI 1.8–23.5) (7). Based on a lower pulsation index observed in infants with hypoplastic left heart syndrome (HLHS), Miller et al. hypothesized that infants with HLHS may have an abnormal systemic vasculature that predisposes them to NEC (8, 9).

These patients remain a challenge for pediatric cardiologists, neonatologists, and intensive care unit personnel. As 48% of NEC develops pre-operatively, physicians are often hesitant to start feeding in infants with duct dependent CHD (10, 11). Nevertheless, there is little evidence showing that pre-operative feeds increase the risk of developing NEC, and most of the evidence comes from retrospective trials (11, 12). Current guidelines recommend that in patients with CHD enteral feeding should be initiated within the preoperative period (13). Despite published recommendations, significant differences in practice exist among centers worldwide (14). This approach contributes to poor growth in the perioperative course (15). Furthermore, adequate nutrition prior to surgery improves postoperative outcomes such as better feeding tolerance, wound healing and length of hospital stay (16).

Increasing evidence supports preoperative feeding in this group of patients despite the increased risk of NEC (5, 17–20). However, only retrospective studies are available, and the relationship between the time of enteral feeding initiation, increasing feeding volume, velocity rates and the development of NEC has not yet been documented (21, 22).

In view of the above, we have decided to perform a prospective randomized study, to evaluate whether implementation of a standard enteral nutrition protocol in patients with duct dependent CHD is equally safe in the context of NEC compared to patients fed based on the discretion of the leading physician.

## Methods and analysis

### Objectives

The aim of this study is to assess whether initiation and advancement of enteral feeding is safe, improves short term outcomes and does not increase the risk of NEC in neonates with duct dependant lesions.

### Trial design

Parents of the neonates born after 36 weeks of pregnancy with duct-dependent CHD will be offered participation in the study within the first 3 days of life. After provision of oral and written information about the study, both parents will be asked to sign a written consent for their child to participate in the study (Table 1).

**Table 1:** Study timetable.

**Timepoint (state unit)**	**T0**	**T1**	**T2**	**T3**	**T4**	**T5**	**T6**	**T7**
**Enrollment**								
Eligibility screen	x							
Informed consent	x							
Allocation	x							
**Interventions**								
	control group	x	x	x	x			
	study group	x	x	x	x			
**Assessments**								
feeding tolerance	x	x	x	x	x			
ECHO assessment	x	x	x	x	x			
Mesenteric and renal artery assessment	x	x	x	x	x			
Anthropometrics weight length head circumference						x	x	x
Breast feeding assessment						x	x	x
**Data collection**								
Baseline demographics	x							
VIS	x	x	x	x				
Protein and caloric intake	x	x	x	x				
days on mechanical ventilation	x	x	x	x				
CLABSI		x	x	x				

CLABSI, central line associated blood stream infection; VIS, vasoactive inotropic support score.

This study is designed as a randomized, parallel-group, superiority multi-center trial, with an 1:1 allocation and stratification by type of lesion.

### Methods: Participants, interventions, and outcomes

#### Study setting

The study will be carried out in four neonatal centers in Poland, including two neonatal departments at the Medical University of Warsaw (MUW), and one Neonatal Unit at Children's Health Memorial Institute (CHMI), and Polish Mother's Memorial Hospital (PMMH).

Departments of Neonatology at MUW are level III and IV teaching hospitals with approximately 5,000 (150 CHD) deliveries per year. Both units are referral centers for fetuses with CHD. Having access to high-risk mothers will augment recruitment. CHMI and PMMH are regional level IV hospital providing neonatal, cardiac, and surgical care for infants with CHD. A certified breastfeeding consultant is available daily on each unit.

The local feeding regimes for infants with CHD are set at the discretion of the attending doctor. However, the staff, in both units, is willing to implement new procedures to improve neonatal outcome.

#### Eligibility criteria

All parents of infants diagnosed with a duct dependant lesion and admitted to participating units will be approached by one of the research team members within the first 72 h of life (as full enteral feeding is usually reached at a minimum of 7 days of life). He or she will explain the study and obtain written consent for participating in the trail. Recruitment will take place between June 1st 2022 and June 30st 2024. After obtaining written consent for participating in the trial, the patient's medical record number (MRN) will be immediately registered on REDCap (Research Electronic Data Capture). Patients eligible for the trial must comply with all the following **at randomization**.

#### Inclusion criteria

1. Duct dependent CHD: assessment of ductus arteriosus (DA) will be done using the parasternal short axis view or suprasternal view.
  - ductal diameter size will be measured using 2D imaging or by color Doppler at the narrowest point usually at the pulmonary end of the duct (mm).
  - shunt direction (using pulsed Doppler) will be defined as one of the followings; left to right or right to left shunt, or bidirectional.
2. Term infants.
3. Parental/legal guardian consent.

#### Exclusion criteria

1. Potential contradictions to early central feeding; neonates with gastrointestinal anatomic and maxillofacial abnormalities.
2. Feeding intolerance defined as vomiting bile or haemorrhagic residuals (23).
3. Hemodynamic instability defined as hypotension requiring at least two inotropes for at least 72 h.
4. > 50% formula based enteral feeding Studies have shown that infants with duct dependent CHD, who were fed with a predominately human milk-based diet have a lower risk of NEC.
5. Birth weight <2500 g In a large multi-center cohort of patients with HLHS, El-Hassan et al. failed to show an increased risk of NEC, but outcomes were worse in the group of babies with low birth weight (24).

Optimal nutrition is crucial to improve short- and long-term outcomes in newborns with CHD. However, large, well designed randomized controlled studies are not available, hence feeding practices differ between units and physicians. Furthermore, a position statement regarding nutritional support for children with critical illnesses (including CHD) is based on studies in preterm populations (13). Given the above, selecting a physician led enteral feeding regime as a comparator is therefore justified.

Patients will be randomized to either the physician led enteral feeding (control group) or protocol-based feeding group (study group).

#### Control group

Patients will receive enteral feeding at the discretion of the leading physician. Feeding practice differs between staff and usually relies on an increase of enteral feeding by 5–20ml/kg/day. Subjective evaluation of feeding tolerance, will be defined as one of the following: bilious or milk residuals, distended abdomen, and vomiting. Withholding or slowing down feeding will be subject to an induvial decision of the attending.

#### Study group

To meet the nutritional goals, infants will receive enteral feeding based on the following protocol (14):

- Minimal enteral nutrition (MEN) will begin within 72 h life at 10 to 20 mL/kg/day, breast milk/donor human milk. MEN will not be included in the caloric goals.
- Advancements in feeding will be set at 20–30 mL/kg/day, but not more than 10 ml per feeding portion to reach a goal of 150 ml/kg/day, but not more than 120ml/kg/day in cases of fluid restriction).
- The goal will be to reach an overall daily caloric intake of minimum 100 kcal/kg/d.

In both groups the interventions will continue up to 28 days of life or day of cardiosurgical treatment, whichever comes first.

### Criteria for modifying or discontinuing allocated interventions

In infants who present with *one or two* of the following symptoms such abdominal distention, visible bowels loops or feeding intolerance (defined as emesis ≥2 consecutive feeds, or gastric residuals of >50% per feed in ≥2 consecutive feeds, bilious residuals, bilious emesis) the intervention will be **modified** by decreasing enteral nutrition by 50% and substituting the remaining fluid requirements with adequate parenteral nutrition for a **maximum** of 48 h. If symptoms persist above 48 h, the trial will be discontinued.

The trial will be **discontinued** if the following occur:

The patient presents with at least three of the following symptoms.

- Abdominal distention.
- Visible bowels loops.
- Feeding intolerance (defined as emesis ≥2 consecutive feeds, or gastric residuals of >50% per feed in ≥2 consecutive feeds, bilious residuals, bilious emesis)..
- Temperature instability (defined as ≥2 consecutive measurements).
- Presence of bowel perforation of NEC confirmed on abdominal x-ray or ultrasound.

### Strategies to improve adherence to interventions

Medical notes (MN) of infants included in the study will be visibly marked to promote adherence to the study protocol. A flowchart explaining inclusion, exclusion and discontinuation criteria will be available in the patient's MN. A meeting will be scheduled to introduce the staff to the study protocol including:

- Brief presentation of the study protocol such as justification for undertaking the trial and possible impact of the study outcome on everyday practice.
- Instructions about the way the intervention should be applied.

Reminder sessions will take place on a two-monthly basis at the departments where the study will be conducted. Staff will be asked about any problems they are experiencing with implementing the study.

### Relevant concomitant care permitted or prohibited during the trial

Participation in clinical trials involving interventions which may bias primary and secondary outcomes results will be prohibited.

### Outcomes

#### Primary outcomes

##### Necrotizing enterocolitis and death related to NEC

The primary outcomes will include Stage II or III of NEC as defined by Kliegman and Walsh (25) and death related to NEC.

Stage II NEC will be defined as distended abdomen and radiological symptoms (intramural and/or portal gas). Stage III NEC will be defined as distended abdomen, radiological symptoms (intramural and/or portal gas) and respiratory and cardiovascular failure requiring mechanical ventilation and inotropic support (25). In surgery verified cases no radiological verification will be required.

#### Secondary outcomes

##### Number of interrupted feedings

Number of interrupted/discontinued feedings per day.

### Growth

Impact on raw and z-score weight, length, and head circumference will be assessed Measurements will be collected at 3, 6 and 12 months of age and reported as z-scores (26).

### Vasoactive support (VA)

Because concomitant use of multiple VA's is common in this group of patients, the vasoactive inotropic support score (VIS) will be calculated to quantify the vasoactive support. The daily VIS will be collected for the complete study period, using the updated definition from Gaies (27). VIS has been previously used to assess enteral feeding safety in a population of critically ill pediatric patients (28). Along the daily scores, the highest VIS and mean VIS will also be calculated.

### Daily protein and caloric intake

Daily intakes of energy (Kcal/kg) and protein (g/kg) provided by enteral and parenteral feeding will be calculated.

### Culture proven late onset sepsis

Defined as sepsis diagnosed >72 h of age, confirmed with a positive blood and/cerebral spinal fluid specimen.

### Days to reach full enteral feeding

The number of days required to reach 150 ml/kg/day of enteral nutrition, counted from day when milk is started.

### Days on mechanical ventilation

The number of days on mechanical ventilation.

### Breastfeeding

Breastfeeding assessment at 3, 6, and 12 months of age. Exclusive breastfeeding will be defined as feeding infants only breast milk, directly from breast or expressed, except drops or syrups consisting of vitamins, mineral supplements, or medicine (29). Partial breastfeeding will be defined as the infant receiving human and formula milk (30).

### Cardiac parameters

Anatomic lesions that disrupt systemic blood flow such as duct dependent lesion, may lead to impaired blood flow which increases the risk of NEC. Carlo et al. demonstrated that, this “diastolic steal phenomenon” appeared in 47% of infants with duct dependent CHD, who developed NEC (31).

However, there are no studies evaluating the mesenteric, aortic, and renal artery blood flow changes after the introduction of feeds.

Doppler assessment will be performed on 1, 3, 7, 14, 28 day of life.

The following measurements will be recorded.

- Peak systolic velocity (PSV) in (cm/s).
- End-diastolic velocity (EDV) in (cm/s).
- Resistivity index (RI).
- Pulsation index (PI).
- Superior mesenteric artery (SMA) blood flow assessment will be performed using the sagittal plane of the abdominal cavity, in the longitudinal projection of the vessel, 1 cm below the celiac trunk.
- Right renal artery (RRA) blood flow assessment will be performed using the frontal plane of the abdominal cavity when the hilum of the kidney is visualized (32).
- direction of diastolic flow in the post-ductal aorta (using pulsed Doppler) will be defined as antegrade, absent or retrograde flow (32).

### Age at transfer

The age (in days) at transfer to a cardiac referral center will recorded, including the length of hospital stay in the referring center, and the reason for transfer.

### Participant timeline

T0- day 1 of life
T1- day 3 of life
T2- day 7 of life
T3-day 14 of life
T4-day 28 of life
T5- 3 months of age
T6 - 6 months of age
T7- 12 months of age.

### Sample size

Studies have reported an incidence of NEC between 7–20% in babies with duct dependent congenital heart diseases, hence we chose the incidence of 18% in the control arm and 7.6% the intervention group (7, 14, 33–35). Therefore, 192 neonates should be included in each group. In total (assuming 20% lost for follow up), 384 neonates with a duct dependent heart disease should be included in the study. These calculations were performed assuming superiority *H0: P1* = *P2* vs. *H1: P1*≥*P2* with a power of 80%, a significance level of 5%. References: https://select-statistics.co.uk/calculators/sample-size-calculator-two-proprtions/ (36).

### Recruitment

The study will continue until the minimum of 192 valid observations are collected in every arm. As part of the antenatal cardiology consult, women with will be offered a short meeting with a member of the recruitment team. During this appointment, they will be offered participation in the trial. To increase participant enrolment a second patient screen will be carried out by medical staff during admission to the Neonatal Intensive Care Unit. The enrolment period will extend over 36 months. Recruitment rates will be monitored monthly. In return, women will be offered additional breastfeeding support.

### Assignment of interventions

#### Allocation (sequence generation, concealment mechanism and implementation)

A member of the recruitment team will approach caregivers within the infant's first 72 h of life. He or she will explain the study and obtain written consent for participating in the trail. Infants who fulfill inclusion criteria will be randomized.

A study number together with the allocated treatment will be assigned by REDCap. The allocation sequence will be computer-generated. Patient's data along with the result of the allocation will be sent to statistical team. Block randomization with stratification by type of lesion (ductal dependent pulmonary, systematic, or mixing blood flow) will be implemented. Patients will be randomly assigned to standard or physician-led enteral nutrition guidelines in 1:1 ratio. To ensure allocation concealment, the randomization list will remain with the statistical team for the whole duration of the study. The block size will be variable and concealed until primary endpoint analyses.

Thus, randomization will be conducted without any influence of the principal investigators, clinicians, recruitment or follow up staff.

### Blinding (masking)

Assessments regarding the clinical course will be conducted by an assessor blind to treatment allocation. Due to the nature of the intervention staff cannot be blinded to allocation but are strongly inculcated not to disclose the allocation status of the participant at the follow up assessments. A member of the research team blinded to treatment allocation will feed data into REDCap.

### Data collection and management

#### Plans for assessment and collection of outcomes

All baseline data will be collected based on electronic MN.

#### Primary outcome

Patients will be evaluated for NEC based on the presence of at least three of the following clinical symptoms (37–40).

- Abdominal distention.
- Visible bowels loops.
- Feeding intolerance (defined as emesis ≥2 consecutive feeds, or gastric residuals of >50% per feed in ≥2 consecutive feeds, bilious residuals, bilious emesis).
- Temperature instability (defined as ≥2 consecutive measurements).
- Frank bloody stools.
- Cardiovascular instability (hypotension; defined as MAP <30 mmHg, tachycardia >160/' or bradycardia <80/').
- Recurrent apnoea.
- Increase of abdominal girth > 2 cm (allowing inter-observer variability of 1 cm) within 12 h
- Abdominal wall erythema.

And/or at least 2 of the below laboratory findings based on Walsh and Bell criteria (modified by Kliegman) (25, 37).

- Thrombocytopenia < 50 k
- Leukopenia <6 k
- CRP > 10 mg/dl
- PCT > 1 IU/l
- Coagulopathy.

NEC will be diagnosed using the abdominal ultrasonography (AUS) or plain abdominal radiography (AR).

Gray- scale AUS will be used to assess bowel wall echogenicity, measure bowel wall thickness, peristalsis, pneumatosis intestinalis (PI), portal vein gas (PVG), free abdominal air and fluid. The following finding on AUS will be equivalent do NEC (41):

1. A bowel wall thickness of >2, 7 mm accompanied by an increase in echogenicity will be considered as suspicious. Color Doppler (CD) ultrasound will be required to evaluate blood flow in the superior mesenteric artery (SMA), and bowel wall perfusion (BWP). Evaluation of BWP; three categories of flow in the bowel wall will be recognized in color Doppler: *normal* (1–9 CD signal dots per cm^2^), *increased* (“zebra” pattern, “Y” pattern or “ring” pattern), and *absent* (when no color Doppler signals will be present) which will be reported as transmural necrosis.
2. Bowel wall thickness below 1.0 mm will be considered as abnormal thinning because of ischaemia or necrosis. Measurements will be recorded in relaxed bowel segments.
3. PI will be defined as small intramural hyperechoic focuses in the bowel wall, which will not change position despite peristalsis, respiratory movement, or abdominal compression with the transducer.
4. Abnormal peristaltic contractions of the small bowel will be defined as <10 movements/min.
5. For evaluation of PVG, the portal vein will be adjusted in a transverse or longitudinal section of the liver. PVG will be defined as highly echogenic round particles with a diameter of approximately 1 mm registered within portal circulation.
6. Using a transverse section of the right upper abdomen showing the liver just below the diaphragm, free abdominal gas will be detected as a bright echogenicity between the abdominal wall and the anterior surface of the liver.
7. Free abdominal fluid will be evaluated as an hypoechogenic material between the bowel loops.

To assess the SMA flow the transducer will be held in the longitudinal section just below the xiphoid. Elevated systolic velocities (up to100–120 cm/s), and high diastolic velocity will be suspicious for NEC.

Ultrasonographer experience will vary between centers (5 to 20 years of experience). All examinations will be performed at the bedside and saved on a secure web-based platform. No specific pre-test requirements will be required.

Plain AR using a vertical and horizontal beam will be repeated every 12–24 h as the disease tends to progress rapidly (42). The following plain abdominal radiography findings will be considered typical for NEC:

- PI is most commonly present in the distal small bowel and large bowel, hence it is usually seen in the right lower quadrant, although it may be present in any part of the gastrointestinal tract (43, 44).
- PVG as an extension of intramural gas present in bowel wall veins, passing into the portal vein system (28, 29).
- *Free intraperitoneal gas* as a result of bowel perforation, which usually is in the distal ileum and proximal colon. It is the only universally accepted radiologic indication for surgical intervention (12).

We will define *AUS as positive for NEC* when three of the following findings are appreciated on examination (41):

1. Abnormal bowel wall thickness (<1.0 mm or >2,7 mm)
2. PI
3. Free abdominal fluid as a focal fluid collection
4. Abnormal SMA blood flow, or bowel wall perfusion.

And one of the listed findings:

1. Free abdominal air
2. Delayed peristalsis
3. PVG.

AR will be classified *as positive for NEC*, if one of the following findings will be present (23):

- Intramural gas (pneumatosis intestinalis)
- Portal venous gas
- Free intraperitoneal gas

*And*

- Bowel dilatation.

#### Staff training

Prior to recruitment commencement, a meeting will be scheduled in each center to introduce the study to unit staff. This session will include:

- Brief presentation on study aims and eligibility criteria.
- Introduction to oral and written informed consent process.
- Presentation of data collection platform and its requirements.
- NEC diagnosis guidelines for Radiologists and Ultrasonographers (if requested).

A subsequent meeting will take place two weeks after initiating the study. Staff will be asked about any problems they might be experiencing with implementing the study, such as patient recruitment, data collection and NEC diagnosis.

Multi-center research meetings will be held two times a year for the first 2 years to improve adherence to study protocol, improve recruitment rates and resolve on-going issues.

All data will be collected on a secure electronic database and will be available on request.

#### Plans to promote participant retention and complete follow-up

All randomized infants who are prematurely discontinued from study drug will be considered *off study intervention/on study*. They will follow the same participant timetable as those infants who continue study intervention. All these infants will be followed through 12 months as scheduled.

Once an infant is enrolled or randomized, the study site will make every reasonable effort to follow the infant for the entire study period. It is projected that the rate of loss-to-follow-up on an annual basis will be at most 20%. Each study site staff will develop and implement local standard operating procedures to achieve this level of follow-up.

#### Data management

Study data will be collected and managed using REDCap (45, 46), an electronic data capture tools hosted at [Princess Anna Mazowiecka's Hospital MUW]. REDCap is a secure, web-based software platform designed to support data capture for research studies, providing (1) an intuitive interface for validated data capture; (2) audit trails for tracking data manipulation and export procedures; (3) automated export procedures for seamless data downloads to common statistical packages; and (4) procedures for data integration and interoperability with external sources (41, 42).

Data integrity will be enforced through a variety of mechanisms. Referential data rules, valid values, range checks, and consistency checks against data already stored in the database (i.e., longitudinal checks) will be supported. Modifications to data written into the database will be documented through either the data change system or an inquiry system. Data entered in the database will be retrievable for viewing through the data entry applications. The type of activity that an individual user may undertake will be regulated by privileges associated with his/her user identification code and password. A complete back up of the database will be performed twice a month.

#### Confidentiality

Complete patient and study information will be stored on REDCap. Only researchers involved in the study will be provided with a personalized login and password to access the study information. The statistical team will not have access to sensitive data such as date of birth, address, contact details. All records containing the above patient details and relevant medical history will be stored separately in a locked file cabinet.

#### Plans for collection, laboratory evaluation and storage of biological specimens for genetic or molecular analysis in this trial/future use

Not applicable.

### Statistical methods

#### Statistical methods for primary and secondary outcomes

As the trial is designed as a superiority study, both primary and secondary outcomes will be analyzed based on the per-protocol (PP) approach.

For primary outcomes and dichotomous secondary outcomes, between-group differences, and the two-sided 95% confidence interval (CI) for the estimated difference approach will be used (47–49).

For continuous outcomes, means/medians and 95% CIs will be calculated. Differences in changes from baseline between treatment groups will be compared using a two-sample t test or Mann-Whitney *U*-test (50).

Additionally, a predictive model to clarify the risk of NEC complications in both study groups will be implemented. Statistical and machine learning techniques such as decision trees, random forests, and support vector machine (SVM) will be used (51–55).

All statistical calculation will be carried out using R package (The R Foundation Vienna, Austria) and Statsoft (Hamburg, Germany).

#### Interim analyses

Our study is a multicentre study, located in 4 different unit. Three interim analyses (IA) will be performed on the primary end point every 100 recruited patients. IA will be conducted by an independent statistician, blinded for the treatment allocation. This will allow us to track trial data collected on case report forms and generate performance metrics. Additionally, it will make data and study information available in a timely manner and re-estimate the sample size if needed. Results of interim analyses will be reported to the DMC, which will decide whether the trial should be continued or stopped for futility based on the pre-specified criteria. The Peto approach will be used: the trial will be ended using symmetric stopping boundaries at *P* < 0.001 (56).

Additionally, sensitivity analysis for the primary outcomes will be carried out with an intention-to-treat (ITT) approach. Protocol deviations will be reviewed by a blinded adjudication DMC who will decide by consensus whether a patient should be included in the ITT or per protocol population. We do not plan any subgroup analyses. In case of missing data, standard data imputation algorithms will be used (e.g., multiple imputation) (55).

#### Plans to give access to the full protocol, participant level-data and statistical code

Access to the full protocol and participation level-data will be given on request from the corresponding author.

## Ethics and dissemination

### Composition of the data monitoring committee (DMC) and trial steering committee (TSC); role and reporting structure

Given the methodology (randomized control trial) and vulnerable population (neonates with congenital heart disease) a Data Monitoring Committee (DMC) has been established. It will be combined of experienced, independent researchers and clinicians (biostatisticians, pediatric cardiologists, pediatric gastroenterologists and neonatologists). The DMC will be independent of the study organizers and will not have financial or intellectual conflict of interest.

It will play an advisory role to the sponsor of the trial, not an executive. It will be up to the sponsor whether to decide or not to accept the DMC's recommendations.

The DMC will monitor the study safety, assess the trial continuing validity and scientific merit. Additionally, it will assess patient recruitment, protocol compliance and data quality.

During the period of recruitment to the study, interim analyses will be supplied, in strict confidence, to the DMC, together with any other analyses that the committee may request. This may include analyses of data from other comparable trials. Considering the interim analysis, the DMC will advise the TSC if, in its view the active intervention has been proved, beyond reasonable doubt, to be different from the control (standard management) for all or some types of participants.

The TSC can then decide whether to modify the trial. Unless this happens, the TSC, clinical collaborators will remain ignorant of the interim results.

The frequency of interim analyses will be carried out every 100 recruited patients, however the schedule may be modified based on depend on the judgement of the Chair of the DMC, in consultation with the TSC.

### Adverse event reporting and harms

We will define an adverse event (AE) as any untoward medical occurrence in a subject without regard to the possibility of a causal relationship. AE will be collected after the subject has provided consent and enrolled in the study. All AE occurring after entry into the study and until hospital discharge will be recorded. An AE that meets the criteria for a serious adverse event (SAE) between study enrolment and hospital discharge will be reported to the local Ethical Committee. A SAE for this study is any untoward medical occurrence that is believed by the investigators to be causally related to study-intervention and results in any of the following: Life-threatening condition (that is, immediate risk of death); severe or permanent disability, prolonged hospitalization. SAE occurring after a subject is discontinued from the study will NOT be reported unless the investigators feels that the event may have been caused by the study drug or a protocol procedure.

### Frequency and plans for auditing trial conduct

We do not plan to audit the trial, unless requested by the DMC.

### Plans for communicating important protocol amendments to relevant parties (e.g., trial participants, ethical committees)

Any modifications to the protocol which may impact on the conduct of the study, potential benefit of the patient or may affect patient safety, including changes of study objectives, study design, patient population, sample sizes, study procedures, or significant administrative aspects will require a formal amendment to the protocol. Such will need to be approved by the Research Ethics Board of the Medical University of Warsaw prior to implementation and notified to the health authorities in accordance with local regulations.

### Dissemination plans

We plan to publish the full protocol, so it shall be widely available due to open access. We plan to submit our findings to international peer-reviewed journals (pediatric, gastroenterology, nutrition). Abstract will be submitted to local and international conferences.

## Discussion

Adequate nutrition is essential to improve short-and long-term outcome in newborns born with CHD. However, significant variation among units worldwide exist in introducing and advancing enteral nutrition in this group of babies. Concerns mainly relate to the risk of feeding intolerance and bowel perforation. Our study will be the first randomized control trial to evaluate if standard (as in healthy newborns) initiation and advancement of enteral feeding is safe, improves short term outcomes and does not increase the risk of NEC. If the studied feeding regime proves to be intact, swift implementation and advancement of enteral nutrition may become a recommendation.

## Ethics statement

The studies involving human participants were reviewed and approved by the Research Ethical Board of the Medical University of Warsaw KB/154/2021. Written, informed consent to participate will be obtained from all participants.

## Author contributions

JS-S conceived the study, led the proposal and protocol development. AP, AW-S, MŻ, JL-G, WZ, MC, and RB contributed to study design and to development of the proposal. KF was the lead trial methodologist. All authors contributed to the article and approved the submitted version.

## Funding

The study will be funded by an internal departmental fund of the Medical University of Warsaw. The funder will not have any role during the study execution, analyses, interpretation of the data or decision to submit results (Grant number: 1W63/1/Z/GW/N/22/22:N).

## Conflict of interest

The authors declare that the research was conducted in the absence of any commercial or financial relationships that could be construed as a potential conflict of interest.

## Publisher's note

All claims expressed in this article are solely those of the authors and do not necessarily represent those of their affiliated organizations, or those of the publisher, the editors and the reviewers. Any product that may be evaluated in this article, or claim that may be made by its manufacturer, is not guaranteed or endorsed by the publisher.

## Abbreviations

NEC: necrotizing enterocolitis
CHD: congenital heart disease
TGA: Transposition of the Great Arteries
TOF: Tetralogy of Fallot
HLHS: Hypoplastic left heart syndrome
MUW: Medical University of Warsaw; CHMI- Children's Health Memorial Institute
MRN: Medical record number
MEN: Minimal enteral nutrition
MN: Medical notes
VIS: Vasoactive inotropes score
VA: vasoactive support
MAP: mean arterial pressure
CRP, C: reactive pressure
PCT: Procalcitonin
AUS: Abdominal ultrasound
CD: Color Doppler
SMA: Superior Mesenteric artery
BWP: Bowel Wall Perfusion
AR: Abdominal radiograph
PVG: Portal vein gas
DMC: Data monitoring committee
CI: Confidence interval
SVM: Support Vector Machine
ITT: intention to treat
TSC: Trial Steering Committee
AE: adverse event
SAE: serious adverse event
CLABSI: central line associated blood stream infection.
